# Giant elephantiasis and inguino-scrotal hernia

**DOI:** 10.1371/journal.pntd.0005494

**Published:** 2017-06-15

**Authors:** Helder Miranda, Anna Claudia Colangelo, Mario Antunes, Marcella Schiavone, Stefano Merigliano, Damiano Pizzol

**Affiliations:** 1 Department of Surgery, Central Hospital of Beira, Beira, Mozambique; 2 Department of Surgery and Organ Transplantation, University of Padua, Padua, Italy; 3 Operational Research Unit, Doctors with Africa Cuamm, Beira, Mozambique; 4 Department of Emergency and Organ Transplantation, Section of Thoracic Surgery, University of Bari, Bari, Italy; Task Force for Child Survival and Development for Global Health, UNITED STATES

## Presentation of the case

**Fig 1:**
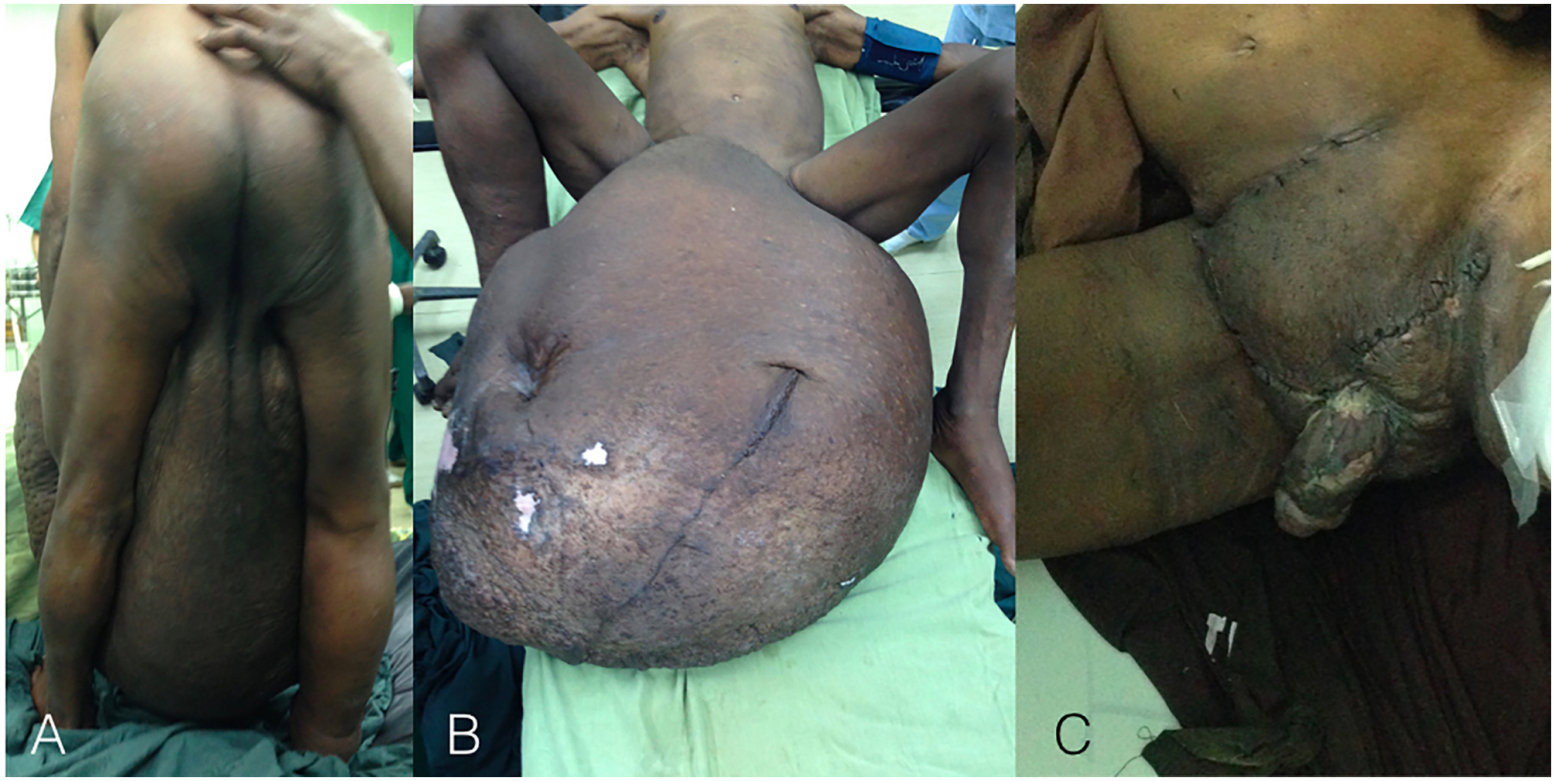
(A) and (B) Preoperative elephantiasis, (C) Postoperative result.

A 65-year-old man presented in Beira Central Hospital, Mozambique, with a right scrotal mass (diameter 80 x 80 cm), evolved over 15 years. The patient could barely move, and his weight at admission was 142 kg (Fig 1A and 1B). History of previous diseases was unremarkable, his general condition was good, and he had normal vital parameters.

Physical examination showed wrinkled and thickened scrotal skin and right leg and foot edema. Due to his physical condition, penile erection had been impossible for many years. The patient was hospitalized for surgery with a diagnosis of “giant elephantiasis of the scrotum with bilateral inguinal hernia”. The man was HIV negative, and preoperative tests showed only a moderate anemia (hemoglobin [Hb] = 7.7 g/dL). The patient was treated with folic acid and multivitamin tablets for 2 months. Finally, he was transfused (4 U), and his Hb increased to 10.2 g/dL before surgery.

Although guidelines suggest hygiene treatment with soap and water for 6 months and antibiotics therapy before surgery, it was not possible to accomplish this protocol, and we proceeded directly with surgery.

Anesthesia was induced intravenously by atropine, 0.5 mg, fentanyl, 150 μg, and thiopental, 500 mg, and it was maintained by fentanyl, 75 μg per hour.

The first step of the surgical procedure was the hydrocele’s reduction, and 15 liters of a brown-colored liquid were aspirated from the mass. After this procedure, there remained a scrotal elephantiasis mass of 67 kg and a bilateral inguino-scrotal hernia. To proceed further with the procedure, it was necessary to do a Foley catheterization in order to get a careful dissection with cautery to delineate the penis circumferentially from the root of the scrotal lymphedema.

A bilateral inguino-scrotal incision was performed. The right testis was stiff and impossible to isolate; the left one was atrophic, and it was not possible to find it. The only solution was to do a bilateral orchiectomy and leave the cords behind in an attempt to form an alternative pathway for lymphatic drainage. The right scrotum presented also a giant inguino-scrotal hernia containing the colon, ileum, and part of the jejunum. The hernia sac was well separated from the internal ring and was easily opened. A hyperemic, inflamed appendix was found; thus, an appendectomy was performed, and the bowels were reduced into the abdomen. The neck of the large hernia sac was transected at the midpoint of the inguinal canal, and the proximal part was sutured—ligated. A high ligation of the proximal sac was done, and the stump was reduced, deep underneath the internal ring. The distal sac was left in place. The hernia repair was finally performed with polypropylene mesh, according to the Lichtenstein tension-free technique. Debulking of lymphedema was done, and the remaining scrotal skin was closed in a Y-shaped manner with the root of penis in the middle (Fig 1B).

During surgery, approximately 2 liters of blood were lost, and 6 units of packed red blood cells and 4 units of fresh frozen plasma were transfused.

Histologic examination showed vascular proliferation, edema, fibrosis, and chronic inflammatory infiltrate.

After 10 days, the patient presented with hypercreatininemia (1.8 mg/dL) and a granulate ulcer of the penis that healed spontaneously. The most important postoperative complication was urethral stenosis, and thus soprapubic cystostomy was performed. The patient was treated with antibiotic therapy for 10 days, and urethral dilatation was performed for 60 days. Eventually, the cystostomy was recovered.

A 6-month follow-up showed a clean scar (Fig 1C) and no sequela, except the erection was not recovered, even after the surgical operation.

## Case discussion

### Inguinal hernia and lymphatic filariasis

WHO estimates that 25 million men experience genital disease [1]. Inguinal hernia and lymphatic filariasis are 2 of the most frequent surgical diseases in Africa [1–2]. Inguinal hernia involves 4.6% of the population in Africa, and males between 20 and 60 years old are the most affected [3]. Over 15 million people are afflicted with lymphedema [1], with multiple etiologies that could be either congenital or acquired (neoplastic, infectious, granulomatous, reactive, disorders of fluid balance, and idiopathic) [4]. Scrotal elephantiasis occurs because of agenesia or hypoplasia, hyperplasia, reflux, overload, or obstruction of the lymphatics [4, 5]. In Mozambique, as in many other countries of sub-Saharan Africa, lymphatic filariasis represents the most important etiology [1]. Lymphatic filariasis is caused by nematode infection belonging to the family Filariodidea: *Wuchereria bancrofti* (responsible for 90% of the cases), *Brugia malayi*, and *B*. *timori* [1]. To guide the surgical management, a standardized clinical classification of hydroceles in lymphatic filariasis was developed based on size of hydrocele (stage I to VI) and on penis burial (grade 0 to 3) [6].

### The presenting case

As a result of the weaknesses of healthcare systems in low- and middle-income countries, extreme presentations of late-stage diseases occur. In Africa, hernia surgery is one of the most frequent procedures done by surgeons, and the high incidence of elephantiasis is explained by filariasis that is endemic in this area. The rarity of our case report is due to 2 factors: the size and the overlap of 3 diseases. On the one hand, the patient presented to us after 15 years of evolving disease, weighing 142 kg before and 70 kg after the surgery. On the other hand, the surgery has resolved 15 liters of hydrocele, giant scrotal lymphedema, and hernia. Although these conditions led to an extreme deformity with ensuing physical, social, and psychological disability, the ineffectiveness of the health system and the lack of trust by the patient had delayed for years the necessary treatment.

The most frequent surgical treatments for genital elephantiasis are as follows: complete excision of all lymphedematous skin and subcutaneous tissue of the penis and scrotum and reconstruction with skin graft, lymphangioplasty, and lymphaticovenous anastomosis. However, our patient presented with chronic fibrosis, and suitable lymphatic channels were not present. For this reason, the excisional surgical repair was the approach required in order to provide the most successful result. While managing the giant inguinal hernia, due to the loss of domain within the abdominal cavity, we reduced the content only with difficulty, fortunately avoiding any respiratory impairment.

## Ethics statement

Written informed consent was obtained from the patient for publication of this case report and any accompanying images.

**Key learning points**

- This case is a rare giant hydrocele (stage IV, grade 3) in scrotal elephantiasis associated with bilateral inguino-scrotal hernia.
- Despite low-resource setting and extreme disease condition, a surgical procedure was performed successfully.
- Due to the high risk of relapse, a regular follow-up is crucial, but in low-resource settings, this rarely happens.
- It is mandatory to strengthen the health system, both in terms of healthcare and of prevention.
